# Fuzzy $H_{\infty}$ output-feedback control for the discrete-time system with channel fadings, sector nonlinearities, and randomly occurring interval delays and nonlinearities

**DOI:** 10.1186/s13662-016-0991-3

**Published:** 2016-10-21

**Authors:** Xiaozheng Fan, Yan Wang, Manfeng Hu

**Affiliations:** 1 School of Science, Jiangnan University, Wuxi, 214122 China; 2 School of Internet of Things, Jiangnan University, Wuxi, 214122 China; 3 Key Laboratory of Advanced Process Control for Light Industry, Ministry of Education, Institute of Automation, Jiangnan University, Wuxi, 214122 China

**Keywords:** Takagi-Sugeno (T-S) fuzzy system, fuzzy $H_{\infty}$ output-feedback control, channel fadings, sector nonlinearities, randomly occurring interval delays (ROIDs), randomly occurring nonlinearities (RONs)

## Abstract

In this paper, the fuzzy $H_{\infty}$ output-feedback control problem is investigated for a class of discrete-time T-S fuzzy systems with channel fadings, sector nonlinearities, randomly occurring interval delays (ROIDs) and randomly occurring nonlinearities (RONs). A series of variables of the randomly occurring phenomena obeying the Bernoulli distribution is used to govern ROIDs and RONs. Meanwhile, the measurement outputs are subject to the sector nonlinearities (*i.e.* the sensor saturations) and we assume the system output is $y(k)=0$, $k\in\{-l,\ldots, 0\}$. The *L*th-order Rice model is utilized to describe the phenomenon of channel fadings by setting different values of the channel coefficients. The aim of this work is to deal with the problem of designing a full-order dynamic fuzzy $H_{\infty}$ output-feedback controller such that the fuzzy closed-loop system is exponentially mean-square stable and the $H_{\infty}$ performance constraint is satisfied, by means of a combination of Lyapunov stability theory and stochastic analysis along with LMI methods. The proposed fuzzy controller parameters are derived by solving a convex optimization problem via the semidefinite programming technique. Finally, a numerical simulation is given to illustrate the feasibility and effectiveness of the proposed design technique.

## Introduction

It is well known that the complexity and nonlinearity of the models are considered as ubiquitous in practical systems. The emergence of this fuzzy modeling approach is based on the Takagi-Sugeno (T-S) fuzzy system (see [1]), which provides a powerful tool for modeling complex nonlinear systems. The $H_{\infty}$ output-feedback control problem for the T-S fuzzy system has received considerable attention (see [2–4]). Nevertheless, the nonlinearity of the T-S fuzzy subsystem is inevitable in practical applications, along with the fact, that the T-S fuzzy model has been successfully used in complex nonlinear systems (see [5–8]). The authors of [8] assumed the nonlinear function in the T-S fuzzy cellular neural networks satisfied the 1-Lipschitz condition and researched the global exponential stability problem for T-S fuzzy cellular neural networks.

In the past decade, networked control systems (NCSs) have played an important role in many engineering applications such as remote militarization, remote medical service, and so on (see [9–11]). However, there are some unavoidable phenomena for the NCSs which may cause poor performance of the controlled systems, for instance, the signal is often transmitted through networks which might be subjected to the occurrence of the phenomenon of incomplete information. The considered incomplete information mainly includes the ROIDs (see [12]), RONs (see [13]), channel fadings (see [14, 15]), and sector nonlinearities (see [16, 17]). The nonlinear output-feedback controller design for polynomial system has been studied (see [18, 19]). A full-order dynamic $H_{\infty}$ output-feedback controller was designed by [20] for the time-varying delays case when all state variables are not available for the feedback. Further, the author of [21] has researched the $H_{\infty}$ output-feedback controller design problem for networked systems with random communication delays by using a linear matrix inequality (LMI) approach. The $H_{\infty}$ output-feedback control problem for a class of discrete-time systems with RONs has been investigated in [22], where random variables are adopted to characterize the RONs and satisfy the binary distribution. The designing $H_{\infty}$ output-feedback controller problems for the T-S fuzzy system with randomly occurring phenomena have been studied in [23, 24]. However, in the case when ROIDs and RONs appear simultaneously in a controlled system, the designing of the fuzzy $H_{\infty}$ output-feedback control problem has received little attention by researchers.

In practical applications, the phenomena of the channel fadings and sector nonlinearities based on unreliable communication networks could occur, which should not be ignored. Considering the situation of signal transmission in fading channels, the $H_{\infty}$ output-feedback control problem with channel fadings has been studied (see [25]). The channel fading has been modeled as a time-varying stochastic model which can describe the transmitted signal’s change in both the amplitude and the phase. The channel fadings with exogenous input disturbance in wireless mobile communications has not been researched extensively (see [12, 22]). On the other hand, the sector nonlinearities of the sensors are usually in order in practical industrial control systems, and this is the main factor that gives rise to the nonlinearity of control systems (see [22]). Since the sensor nonlinearity cannot be neglected and often leads to bad performance of the discrete-time control system, it has attracted the attention of researchers (see [26–28]).

In [22], the $H_{\infty}$ output-feedback control problem for a class of discrete-time systems with channel fadings and sector nonlinearities has been studied, and the existence of the desired controllers has been derived via using a combination of the stochastic analysis and Lyapunov function approach. The design of $H_{\infty}$ fuzzy controller problem for the fuzzy system with the probabilistic infinite-distributed delay and the channel fadings also has been investigated in [23], where the channel fading model can better reflect the reality of measurement transmission especially through a wireless sensor network. So far, to the best of the authors’ knowledge, the fuzzy $H_{\infty}$ output-feedback control problem for a class of discrete-time T-S fuzzy system with channel fadings, sector nonlinearities, ROIDs and RONs have not been investigated yet, and the main purpose of this paper is to bridge such a gap.

The main contributions of this paper are summarized as follows. (1) Both the probabilistic interval time-varying delays and the randomly occurring nonlinearities are used for describing the discrete-time fuzzy model. (2) Moreover, a newly fuzzy control system as well as measurement model is put forward which can account for the randomly occurring incomplete information phenomena of the sensor saturation and the channel fadings. (3) A new fuzzy $H_{\infty}$ output-feedback controller has been designed.

Motivated by the above discussion, this paper intends to study the fuzzy $H_{\infty}$ output-feedback control problem for a class of discrete-time Takagi-Sugeno (T-S) fuzzy model system with channel fadings, sector nonlinearities, ROIDs, and RONs. The rest of this paper is organized as follows. In the next section, the problem descriptions of the discrete-time T-S fuzzy system with ROIDs, RONs, sector nonlinearities, and channel fadings are stated, and the necessary definitions and relevant lemmas are recalled. Section 3 presents the main results of this paper. Illustrative examples are provided in Section 4. Finally, conclusions are drawn in Section 5.

## Model description and preliminaries

In this paper, we consider the following discrete-time fuzzy system with RONs and ROIDs is described by the following fuzzy **IF-THEN** rules:

Plant Rule i: **IF** $\theta_{1}(k)$ is $\mathcal{M}_{i1}$ and $\theta _{2}(k)$ is $\mathcal{M}_{i2},\ldots$ , and $\theta_{r}(k)$ is $\mathcal{M}_{ir}$, **THEN**

1 $$ \left \{ \textstyle\begin{array}{@{}l} x(k+1)=A_{1i}x(k)+A_{2i}\sum_{m}^{h}\beta_{m}(k)x(k-\tau_{m}(k))+B_{i}u(k) +D_{1i}\omega(k)+r(k)f(x(k)),\\ z(k)=E_{i}x(k)+D_{2i}\omega(k),\\ x(s)=\phi(s),\quad s=-h,-h +1,\ldots,1,0, \end{array}\displaystyle \right . $$

where $i=1,\ldots,r$, the system (1) is equivalent to a fuzzy combination of *r* subsystems. $\mathcal{M}_{ij}$ is the fuzzy set, $\theta(k)=[\theta_{1}(k), \theta_{2}(k), \ldots, \theta_{r}(k)]$ is the premise variable vector. $x(k)\in\mathbb{R}^{n}$ is the state vector with $x(0)=\phi (0)$, $u(k)\in \mathbb{R}^{r}$ is the control input vector, $z(k)\in\mathbb{R}^{q}$ is the controlled output vector, $\omega(k)\in l_{2}([0,+\infty),\mathbb {R}^{n})$ is the exogenous disturbance input. $\phi(s)$ is the initial state. $A_{1i}, A_{2i}, B_{i}, D_{1i}, E_{i}$, and $D_{2i}$ are known real matrices with appropriate dimensions.

To characterize the phenomena of randomly occurring interval delays and randomly occurring nonlinearities, we employ the stochastic variables $\beta_{m}(k)$ ($m=1,\ldots,h$) and $r(k)$ in (1), which are mutually independent Bernoulli-distributed white sequences with the following probability distribution:

$$\begin{aligned}& \operatorname{Prob}\bigl\{ \beta_{m}(k)=1\bigr\} =\mathbb{E}\bigl\{ \beta_{m}(k)\bigr\} =\bar{\beta}_{m}, \\& \operatorname{Prob}\bigl\{ \beta_{m}(k)=0\bigr\} =1-\bar{ \beta}_{m}, \\& \operatorname{Prob}\bigl\{ r(k)=1\bigr\} =\mathbb{E}\bigl\{ r(k)\bigr\} =\bar{r}, \\& \operatorname{Prob}\bigl\{ r(k)=0\bigr\} =1-\bar{r}. \end{aligned}$$

The variable $\tau_{m}(k)$ ($m=1,2,\ldots,h$) means the time-varying delays satisfying

2 $$ \underline{d}_{m}\leq\tau_{m}(k)\leq \bar{d}_{m}, $$

where $\underline{d}_{m}$ and $\bar{d}_{m}$ are real positive integers representing the lower bounds and the upper bounds on the communication delay, respectively.

### Assumption 1

The nonlinear vector-valued function $f(x(k)):\mathbb{R}^{n}\rightarrow \mathbb{R}^{n}$ with $f(0)=0$ is seen as continuous and satisfies the following sector-bounded condition:

3 $$ \bigl\Vert f\bigl(x(k)\bigr)\bigr\Vert ^{2}\leq \lambda\bigl\| Gx(k)\bigr\| ^{2} $$

for all $k\in[0,N]$, where $\lambda>0$ is a known positive scalar and *G* is a known real matrix.

### Remark 1

In model (1), the stochastic variables $r(k)$ is used for characterizing the phenomena of RONs. The T-S fuzzy model with RONs includes the fuzzy model with nonlinearity in [6, 8] as a special case where the values of $r(k)$ are 1. Note that RONs is considered for the first time for fuzzy output-feedback control problem. On the other hand, the occurrence of the ROIDs $x(k-\tau_{m}(k))$ is characterized by the random variables $\beta_{m}(k)$ in a probabilistic way, which is more suitable for reflecting the network-induced phenomena. Meanwhile, it is worth of note that there are some results concerned with the continuous time-varying delays in [29, 30] and few results for randomly occurring interval delays, especially when the fuzzy $H_{\infty}$ output-feedback control problem becomes a research focus.

Let us now consider the case when the phenomena of the sector nonlinearities and the channel fadings may occur in signal transmission, where the system output is subject to sector-like bounds and the sensor signal sent to the actuator subject to channel fadings for the control purpose. The signal received by the actuator is modeled in the following:

4 $$ \left \{ \textstyle\begin{array}{@{}l} y(k)=g(x(k)),\\ \xi(k)=\sum_{l=0}^{\ell}\alpha_{l}(k)y(k-l)+\nu(k), \end{array}\displaystyle \right . $$

where $y(k)\in\mathbb{R}^{n}$ represents the system output with $y(k)=0,\forall k\in\{-l, \ldots,0\}$, $g(x(k))$ is the sector nonlinearity of the sensor, $\xi(k)\in\mathbb{R}^{n}$ is the signal from the actuator, and $\nu(k)\in l_{2}([0,+\infty),\mathbb{R}^{n})$ is an external disturbance. $\alpha_{l}(k)\in\mathbb{R}$ ($l=0,1,\ldots,\ell$) is the channel coefficient which is independent and conform Gaussian random variables distributed with mean $\bar{\alpha}_{l}$ and variance $\tilde{\alpha}_{l}^{2}$. In practice, the channel coefficients typically take values over the interval $[0,1]$.

### Assumption 2

The nonlinear function $g(x(k))$ in (4) represents the sector nonlinearities satisfying the following sector condition:

5 $$ \bigl(g\bigl(x(k)\bigr)-M_{1}x(k)\bigr)^{T} \bigl(g\bigl(x(k)\bigr)-M_{2}x(k)\bigr)\leq0, $$

where $M_{1}$ and $M_{2}$ ($M_{2}>M_{1}\geq0$) are known real matrices with appropriate dimensions.

### Remark 2

In this paper, the channel fadings and sector nonlinearities of the sensors can be described simultaneously by the model (4) in the measurement. In [31], this Rice fadings model can properly describe the phenomena of the channel fadings, time-delay, and date dropout, therefore the fadings model can be employed in this paper for the design of the $H_{\infty}$ fuzzy output-feedback controller of the discrete-time fuzzy system.

### Remark 3

The sector nonlinearities of the sensors usually occur in practical network control systems and cause poor performance of the controlled system. The analysis and synthesis problems for a series of dynamics systems with sector nonlinearities has been investigated in [17, 32]. Particularly, the nonlinear function lies inside the sector $[M_{1}, M_{2}]$ in [17]. Furthermore, the Lipschitz condition can be concluded by the nonlinear description as a special case if $M_{1}=0$ or $M_{2}=0$ in Assumption 2.

### Assumption 3

[22]

For technical convenience, the nonlinear function $g(x(k))$ can be decomposed into a linear and a nonlinear parts as

6 $$ g\bigl(x(k)\bigr)=g_{s}\bigl(x(k)\bigr)+M_{1}x(k), $$

where the nonlinear part $g_{s}(x(k))$ belongs to the set $G_{s}$ defined by

7 $$ G_{s}=\bigl\{ g_{s}\bigl(x(k) \bigr):g_{s}^{T}\bigl(x(k)\bigr) \bigl(g_{s} \bigl(x(k)\bigr)-Mx(k)\bigr)\leq0\bigr\} $$

with $M=M_{1}-M_{2}>0$.

In this paper, we adopt a full-order fuzzy output-feedback controller for the discrete-time system by the fuzzy **IF-THEN** rules as follows:

Control rule i: **IF** $\theta_{1}(k)$ is $\mathcal{M}_{i1}$ and $\theta _{2}(k)$ is $\mathcal{M}_{i2},\ldots$ , and $\theta_{r}(k)$ is $\mathcal{M}_{ir}$, **THEN**

8 $$ \left \{ \textstyle\begin{array}{@{}l} x_{c}(k+1)=A_{ci}x_{c}(k)+B_{ci}\xi(k),\\ u(k)=C_{ci}x_{c}(k), \end{array}\displaystyle \right . $$

where $x_{c}(k)\in\mathbb{R}^{n}$ is the state estimate of system (1), and $A_{ci}, B_{ci}, C_{ci}$ are appropriately dimensioned parameters matrices to be determined.

Set

9 $$ h_{i}\bigl(\theta_{i}(k)\bigr)= \frac{\prod_{j=1}^{r}\mathcal{M}_{ij}(\theta _{j}(k))}{\sum_{i=1}^{r}\prod_{j=1}^{r}\mathcal{M}_{ij}(\theta_{j}(k))}, $$

where $\mathcal{M}_{ij}(\theta_{j}(k))$ denotes the grade of membership of $\theta_{j}(k)$ in $\mathcal{M}_{ij}$. Obviously, $h_{i}(\theta(k))\geq 0$, and $\sum_{i=1}^{r}h_{i}(\theta(k))=1$. To ease the presentation, we use $h_{i}$ instead of $h_{i}(\theta(k))$.

Above all, the T-S fuzzy system (1) model can be constructed as follows:

10 $$ \left \{ \textstyle\begin{array}{@{}l} x(k+1)=\sum_{i=1}^{r}h_{i}\{A_{1i}x(k)+A_{2i}\sum_{m}^{h}\beta_{m}(k)x(k-\tau _{m}(k))+B_{i}u(k)\\ \hphantom{x(k+1)=}{}+D_{1i}\omega(k)+r(k)f(x(k))\},\\ z(k)=\sum_{i=1}^{r}h_{i}\{E_{i}x(k)+D_{2i}\omega(k)\}. \end{array}\displaystyle \right . $$

Furthermore, the fuzzy control system can be described by

11 $$ \left \{ \textstyle\begin{array}{@{}l} x_{c}(k+1)=\sum_{i=1}^{r}h_{i}\{A_{ci}x_{c}(k)+B_{ci}\xi(k)\},\\ u(k) =\sum_{i=1}^{r}h_{i}C_{ci}x_{c}(k). \end{array}\displaystyle \right . $$

Combining (4), (6), (10), and (11), the fuzzy control system can be represented by

12 $$ \left \{ \textstyle\begin{array}{@{}l} \eta(k+1)=\sum_{i=1}^{r}\sum_{j=1}^{r}h_{i}h_{j}\{\bar{A}_{1ij}\eta (k)+\bar{A}_{2i}\sum_{m=1}^{h}\bar{\beta}_{m}Z\eta(k-\tau_{m}(k))\\ \hphantom{\eta(k+1)=}{}+ \bar{A}_{2i}\sum_{m=1}^{h}\tilde{\beta}_{m}(k)Z\eta(k-\tau_{m}(k))+\mathcal {D}_{1ij}\tilde{\omega}(k)+\bar{r}\mathcal{F}(\eta(k))+\tilde {r}(k)\mathcal{F}(\eta(k))\\ \hphantom{\eta(k+1)=}{} +\sum_{l=1}^{\ell}\bar{\alpha}_{l}\bar{B}_{j}Z\eta(k-l)+\sum_{l=0}^{\ell }\tilde{\alpha}_{l}(k)\bar{B}_{(}j)Z\eta(k-l)\\ \hphantom{\eta(k+1)=}{}+\sum_{l=0}^{\ell}\bar{\alpha}_{l}\tilde {B}_{j}g_{v}(Z\eta(k-l)) +\sum_{l=0}^{\ell}\tilde{\alpha}_{l}(k)\tilde{B}_{j}g_{v}(Z\eta(k-l))\} ,\\ z_{k}=\sum_{i=1}^{r}\sum_{j=1}^{r}h_{i}h_{j}\{\mathcal{E}_{i}\eta (k)+\mathcal{D}_{2j}\tilde{\omega}(k)\}, \end{array}\displaystyle \right . $$

where

$$\begin{aligned}& \eta(k)=\bigl(x^{T}(k),x_{c}^{T}(k) \bigr)^{T},\qquad\tilde{\omega}=\bigl(\omega^{T}(k), \nu^{T}(k)\bigr)^{T},\qquad\mathcal{F}\bigl(\eta(k)\bigr)= \begin{bmatrix} f(x(k))\\ 0 \end{bmatrix} , \\& \bar{A}_{1ij}= \begin{bmatrix} A_{1i} & B_{i}C_{cj}\\ \bar{\alpha}_{0}B_{cj}M_{1}& A_{cj} \end{bmatrix} , \qquad\bar{A}_{2i}= \begin{bmatrix} A_{2i}\\ 0 \end{bmatrix} ,\qquad Z= [ \textstyle\begin{array}{@{}c@{\quad}c@{}} I_{n} & 0 \end{array}\displaystyle ], \\& \bar{B}_{j}= \begin{bmatrix} 0 \\ B_{cj}M_{1} \end{bmatrix} , \qquad\tilde{B}_{j}= \begin{bmatrix} 0 \\ B_{cj} \end{bmatrix} , \qquad \mathcal{E}_{i}=[ \textstyle\begin{array}{@{}c@{\quad}c@{}} E_{i} & 0 \end{array}\displaystyle ], \\& \mathcal{D}_{1ij}= \begin{bmatrix} D_{1i} & 0\\ o & B_{cj} \end{bmatrix} , \qquad\mathcal{D}_{2j}= [ \textstyle\begin{array}{@{}c@{\quad}c@{}} D_{2j} & 0 \end{array}\displaystyle ], \end{aligned}$$

with

$$\begin{aligned}& \tilde{\alpha}(k)=\alpha(k)-\bar{\alpha}; \\& \tilde{\beta}_{m}(k)=\beta_{m}(k)-\bar{ \beta}_{m}; \\& \tilde{r}(k)=r(k)-\bar{r}. \end{aligned}$$

Obviously

$$\begin{aligned}& \mathbb{E}\bigl\{ \tilde{\alpha}(k)\bigr\} =0,\qquad\mathbb{E}\bigl\{ \tilde{ \alpha}^{2}(k)\bigr\} \triangleq\tilde{\alpha}_{l}^{2} \quad(l=0,1,\ldots,\ell); \\& \mathbb{E}\bigl\{ \tilde{\beta}_{m}(k)\bigr\} =0,\qquad\mathbb{E}\bigl\{ \tilde{\beta}_{m}^{2}(k)\bigr\} =\bar{\beta}_{m}(1- \bar{\beta}_{m})\triangleq\tilde{\beta}_{m}^{2} \quad(m=1,\ldots,h); \\& \mathbb{E}\bigl\{ \tilde{r}(k)\bigr\} =0,\qquad\mathbb{E}\bigl\{ \tilde{r}^{2}(k)\bigr\} =\bar{r}(1-\bar{r})\triangleq \tilde{r}^{2}. \end{aligned}$$

Particularly, we can see from Assumption 1 that the nonlinear function $\mathcal{F}(\eta(k))$ satisfies the following formula:

13 $$ \mathcal{F}^{T}\bigl(\eta(k)\bigr)\mathcal{F}\bigl( \eta(k)\bigr)\leq\lambda\eta^{T}(k)\mathcal{G}^{T}\mathcal{G} \eta(k), $$

where $\mathcal{G}=(G,0)$.

To describe our main result more precisely, we first introduce the following definition and lemmas.

### Definition 1

Exponentially mean-square stability [33]

The T-S fuzzy control system with channel fadings in (12) and every initial conditions *ϕ*, the zero solution is said to be exponentially mean-square stable if, in the case of $\tilde{\omega }(k)=0$, then exist constants $\delta>0 $ and $0<\varrho<1$ such that

14 $$ \mathbb{E}\bigl\{ \bigl\Vert \eta(k)\bigr\Vert ^{2} \bigr\} \leq\delta\varrho^{k}\sup_{i\in\mathbb {K}}\mathbb{E}\bigl\{ \bigl\Vert \phi(i)\bigr\Vert ^{2}\bigr\} ,\quad\forall k\geq0. $$

With Definition 1, the aim of this paper is to design a robust $H_{\infty}$ output-feedback controller in the form of (11) such that the fuzzy discrete-time system (12) is exponentially mean-square stable and the $H_{\infty}$ performance is satisfied or, more specifically, the following two requirements are satisfied simultaneously:

(R1) The fuzzy discrete-time system (12) is exponentially mean-square stable.

(R2) Under the zero-initial condition, the controlled output $z(k)$ satisfies

15 $$ \sum_{k=0}^{\infty}\mathbb{E} \bigl\{ \bigl\Vert z(k)\bigr\Vert ^{2}\bigr\} \leq \gamma^{2}\sum_{k=0}^{\infty}\mathbb{E} \bigl\{ \bigl\Vert \tilde{\omega}(k)\bigr\Vert ^{2}\bigr\} $$

for all nonzero $\tilde{\omega}(k)$, where $\gamma>0$ is a prescribed scalar.

### Lemma 1

Schur complement [34]

*We have the linear matrix inequality*

$$S= \begin{bmatrix} S_{11} & S_{12} \\ S_{12}^{T} & S_{22} \end{bmatrix} < 0, $$

*where* $S_{11}=S_{11}^{T}$ *and* $S_{22}=S_{22}^{T}$ *are equivalent to*

$$S_{11}< 0,\qquad S_{22}-S_{12}^{T}S_{11}^{-1}S_{12}< 0 . $$

### Lemma 2

[35]

*For a symmetric positive definite* *S*, *and any real matrices* $X_{(ijp)}$ *with appropriate dimensions*, *we can get*

$$\begin{aligned} &\sum_{i=1}^{r}\sum _{j=1}^{r}\sum_{s=1}^{r} \sum_{t=1}^{r}h_{i}h_{j}h_{s}h_{t} (X_{(ij)})^{T}SX_{(st)} \leq\sum_{i=1}^{r}\sum _{j=1}^{r}h_{i}h_{j}(X_{(ij)})^{T}SX_{(ij)}. \end{aligned}$$

### Lemma 3

[36]

*Given any matrices* $x,y$, *a matrix* $P>0$, *and a positive scalar* *ϵ*, *then we have*

$$2x^{T}y\leq\epsilon x^{T}Px+\epsilon^{-1} y^{T}Py. $$

## Main result

In this part, the following theorem provides a sufficient condition for the discrete-time T-S fuzzy system (12) to be exponentially mean-square stable and the controlled output $z_{k}$ to satisfy the $H_{\infty}$ disturbance reject requirement in (15).

### Theorem 1

*Let a scalar* $\gamma>0$ *and the controller parameters matrix* $A_{cj}, B_{cj}$, *and* $C_{cj}$ ($j=1,\ldots,r$) *be given*. *The fuzzy closed*-*loop system* (12) *is exponentially men*-*square stable and the controlled output* $z(k)$ *satisfies* (15), *if there exist matrices* $P>0,Q_{m}>0$, *and* $R_{l}>0$ ($m=1,\ldots,h$; $l=0,1,\ldots,\ell$), *and a positive scalar* $\psi >0 $ *and* $\varphi>0$ *satisfying*

16 $$\begin{aligned}& \Upsilon_{ii}^{1}< 0, \end{aligned}$$

17 $$\begin{aligned}& 2\bigl(\Upsilon_{ij}^{1}+\Upsilon_{ji}^{1} \bigr)< 0, \end{aligned}$$

*where* $1\leq i < j \leq r$ ($i,j\in R$)

$$\begin{aligned}& \Upsilon_{ij}^{1}= \begin{bmatrix} \varLambda _{11}^{1} & \varOmega _{12ij}^{1} & 0\\ * & \varLambda _{22^{1}} & \varOmega _{23ii}^{1}\\ * & * & \varLambda _{33}^{1} \end{bmatrix} , \\& \varLambda _{11}^{1}=\operatorname{diag}\{-P, - \mathcal{P}_{\ell+1}, -I, -\varphi I \},\qquad\mathcal{P}_{s}= \operatorname{diag}\{\underbrace{P,\ldots,P}_{s}\}, \\& \varLambda _{22}^{1}=\operatorname{diag}\bigl\{ -P, - \mathcal{Q}_{h}, -\mathcal{R}_{\ell}, -\varphi I, -\psi I, - \gamma^{2} I\bigr\} , \\& \varLambda _{33}^{1}=\operatorname{diag}\{- \mathcal{P}_{h}, -P, -\bar{\mathcal{Q}}_{m}, - \mathcal{R}_{\ell}, -\psi I_{\ell+1}\},\qquad-\bar{ \mathcal{Q}}_{m}=\sum_{m=1}^{h}( \bar{d}_{m}-\underline{d}_{m}+1)Z^{T}Q_{m} Z, \\& \varOmega _{12ij}^{1}= \begin{bmatrix} P\bar{A}_{1ij} & P(\bar{\Theta}_{1}\otimes\bar{A}_{2i}) & P(\bar{\Theta }_{2}\otimes\bar{B}_{j}) & \bar{r}P & P(\bar{\Theta}_{3}\otimes\tilde {B}_{j}) & P\mathcal{D}_{1ij})\\ 0 & 0 & \mathcal{P}_{\ell+1}(\tilde{\Theta}_{3}\otimes\bar{B}_{j}) & 0 & \mathcal{P}_{\ell+1}(\tilde{\Theta}_{3}\otimes\tilde{B}_{j}) & 0\\ \mathcal{E}_{i} & 0 & 0 & 0 & 0 & \mathcal{D}_{2j}\\ \varphi\sqrt{\lambda}\mathcal{G} & 0 & 0 & 0 & 0 & 0 \end{bmatrix} , \\& \varOmega _{23ii}^{1}= \begin{bmatrix} 0 & 0 & Z^{T} & \mathcal{Z}_{\ell}^{T} & 0\\ (\tilde{\Theta}_{1}\otimes\bar{A}_{2i}^{T})\mathcal{P}_{h} & 0 & 0 & 0 & 0\\ 0 & 0 & 0 & 0 & \psi\mathcal{M}^{T}\\ 0 & \tilde{r}P & 0 & 0 & 0\\ 0 & 0 & 0 & 0 & 0\\ 0 & 0 & 0 & 0 & 0 \end{bmatrix} ^{T} , \\& \mathcal{Q}_{h}=\operatorname{diag}\{Q_{1}, \ldots,Q_{h}\},\qquad\mathcal{R}_{\ell}=\operatorname{diag} \{I,R_{1},\ldots,R_{\ell}\}, \\& \mathcal{M}=\operatorname{diag}\{\underbrace{M,\ldots,M}_{\ell+1}\} ,\qquad I_{s}=\operatorname{diag}\{\underbrace{I,\ldots,I}_{s}\}, \qquad\mathcal{Z}_{\ell}=\operatorname{diag}\bigl\{ \underbrace{Z^{T}, \ldots,Z^{T}}_{\ell+1}\bigr\} ^{T}, \\& \bar{\Theta}_{1}=(\bar{\beta}_{1},\ldots,\bar{ \beta}_{h}),\qquad\tilde{\Theta}_{1}=\operatorname{diag}\{ \tilde{\beta}_{1},\ldots,\tilde{\beta}_{h}\}, \\& \bar{\Theta}_{2}=(0,\bar{\alpha}_{1},\ldots,\bar{ \alpha}_{\ell}),\qquad\bar{\Theta}_{3}=(\bar{ \alpha}_{0},\bar{\alpha}_{1},\ldots,\bar{\alpha }_{\ell}), \\& \tilde{\Theta}_{3}=\operatorname{diag}\{\tilde{\alpha}_{0}, \tilde{\alpha}_{1},\ldots,\tilde{\alpha}_{\ell}\}. \end{aligned}$$

### Proof

We choose the following Lyapunov function:

$$V\bigl(x(k)\bigr)=\sum_{i=1}^{4}V_{i} \bigl(x(k)\bigr), $$

where

18 $$\begin{aligned} &V_{1}(k)=\eta^{T}(k)P\eta(k), \\ &V_{2}(k)=\sum_{m=1}^{h}\sum _{i=k-\tau_{m}(k)}^{k-1}\eta^{T}(i)Z^{T}Q_{m}Z \eta(i),\\ &V_{3}(k)=\sum_{m=1}^{h}\sum _{n=-\bar{d}_{m}+1}^{-\underline{d}_{m}} \sum _{i=k+n}^{k-1}\eta^{T}(i)Z^{T}Q_{m}Z \eta(i), \\ &V_{4}(k)=\sum_{l=1}^{\ell}\sum _{i=k-l}^{k-1}\eta^{T}(i)Z^{T} \mathcal{R}_{l}Z\eta(i). \end{aligned}$$

The difference of $V(x(k))$ along the trajectory of the system (12) is

19 $$\begin{aligned} \mathbb{E}\bigl\{ \Delta V(k)\bigr\} &=\mathbb{E} \bigl\{ V\bigl(x(k+1)\bigr)-V\bigl(x(k)\bigr)\bigr\} =\sum_{i=1}^{4}\mathbb{E}\bigl\{ V_{i}\bigl(x(k+1)\bigr)- V_{i}\bigl(x(k)\bigr)\bigr\} . \end{aligned}$$

We have

$$\begin{aligned} \mathbb{E}\bigl\{ \Delta V_{1}(k)\bigr\} ={}&\mathbb{E}\bigl\{ V_{1}\bigl(x(k+1)\bigr)-V_{1}\bigl(x(k)\bigr)\bigr\} =\mathbb{E}\bigl\{ \eta_{k+1}^{T}P\eta_{k+1}- \eta_{k}^{T}P\eta_{k}\bigr\} \\ ={}&\mathbb{E}\Biggl\{ \sum_{i=1}^{r}\sum _{j=1}^{r}\sum_{s=1}^{r} \sum_{t=1}^{n}h_{i}h_{j}h_{s}h_{t} \Biggl[\bar{A}_{1ij}\eta(k)+\bar{A}_{2i}\sum_{m=1}^{h} \bar{\beta}_{m}Z\eta\bigl(k-\tau_{m}(k)\bigr) \\ &{}+\bar {A}_{2i}\sum_{m=1}^{h}\tilde{ \beta}_{m}(k)Z\eta\bigl(k-\tau_{m}(k)\bigr) +\mathcal{D}_{1ij}\tilde{\omega}(k)+\bar{r}\mathcal{F}\bigl(\eta(k) \bigr)+\tilde{r}(k)\mathcal{F}\bigl(\eta(k)\bigr) \\ &{}+\sum _{l=1}^{\ell}\bar{\alpha}_{l} \bar{B}_{j}Z\eta(k-l) +\sum_{l=0}^{\ell}\tilde{ \alpha}_{l}(k)\bar{B}_{(}j)Z\eta(k-l) +\sum_{l=0}^{\ell} \bar{\alpha}_{l}\tilde{B}_{j}g_{v}\bigl(Z \eta(k-l)\bigr) \\ &{} +\sum_{l=0}^{\ell}\tilde{ \alpha}_{l}(k)\tilde{B}_{j}g_{v}\bigl(Z\eta(k-l) \bigr)\Biggr]^{T}P\Biggl[\bar{A}_{1st}\eta(k)+\bar{A}_{2s}\sum _{m=1}^{h}\bar{\beta}_{m}Z\eta \bigl(k-\tau_{m}(k)\bigr) \\ &{} +\bar{A}_{2s}\sum _{m=1}^{h}\tilde{\beta}_{m}(k)Z\eta\bigl(k- \tau_{m}(k)\bigr) +\mathcal{D}_{1st}\tilde{\omega}(k)+\bar{r}\mathcal{F}\bigl(\eta(k) \bigr)+\tilde{r}(k)\mathcal{F}\bigl(\eta(k)\bigr)\\ &{} +\sum _{l=1}^{\ell}\bar{\alpha}_{l} \bar{B}_{t}Z\eta(k-l) +\sum_{l=0}^{\ell}\tilde{ \alpha}_{l}(k)\bar{B}_{t}Z\eta(k-l) +\sum _{l=0}^{\ell}\bar{\alpha}_{l} \tilde{B}_{t}g_{v}\bigl(Z\eta(k-l)\bigr) \\ &{}+\sum_{l=0}^{\ell}\tilde{ \alpha}_{l}(k)\tilde{B}_{t}g_{v}\bigl(Z\eta(k-l) \bigr)\Biggr]-\eta(k)^{T}P\eta(k)\Biggr\} \\ ={}&\mathbb{E}\Biggl\{ \sum_{i=1}^{r}\sum _{j=1}^{r}\sum_{s=1}^{r} \sum_{t=1}^{n}h_{i}h_{j}h_{s}h_{t} \Biggl[\eta(k)^{T}\bar{A}_{1ij}^{T}P\bar {A}_{1st}\eta(k) \\ &{}+2\eta(k)^{T}\bar{A}_{1ij}^{T}P \bar{A}_{2s}\sum_{m=1}^{h}\bar{ \beta}_{m}Z\eta\bigl(k-\tau_{m}(k)\bigr) \\ &{}+2\eta(k)^{T}\bar{A}_{1ij}^{T}P \mathcal{D}_{1st}\tilde{\omega}(k) +2\eta(k)^{T} \bar{A}_{1ij}^{T}P\bar{r}\mathcal{F}\bigl(\eta(k)\bigr) \\ &{}+2\eta(k)^{T}\bar{A}_{1ij}^{T}P\sum _{l=1}^{\ell}\bar{\alpha}_{l}\bar {B}_{t}Z\eta(k-l) +2\eta(k)^{T}\bar{A}_{1ij}^{T}P\sum _{l=0}^{\ell}\bar{\alpha}_{l}\tilde {B}_{t}g_{v}\bigl(Z\eta(k-l)\bigr) \\ &{}+\sum_{m=1}^{h}\bar{ \beta}_{m}Z\eta^{T}\bigl(k-\tau_{m}(k)\bigr) \bar{A}_{2i}^{T}P\bar{A}_{2s}\sum _{m=1}^{h}\bar{\beta}_{m}Z\eta\bigl(k- \tau_{m}(k)\bigr) \\ &{}+2\sum_{m=1}^{h}\bar{ \beta}_{m}Z\eta^{T}\bigl(k-\tau_{m}(k)\bigr) \bar{A}_{2i}^{T}P\mathcal{D}_{1st}\tilde{\omega}(k) \\ &{}+2\sum_{m=1}^{h}\bar{ \beta}_{m}Z\eta^{T}\bigl(k-\tau_{m}(k)\bigr) \bar{A}_{2i}^{T}P\bar{r}\mathcal{F}\bigl(\eta(k)\bigr) \\ &{}+2\sum_{m=1}^{h}\bar{ \beta}_{m}Z\eta^{T}\bigl(k-\tau_{m}(k)\bigr) \bar{A}_{2i}^{T}P\sum_{l=1}^{\ell} \bar{\alpha}_{l}\bar{B}_{t}Z\eta(k-l) \\ &{}+2\sum_{m=1}^{h}\bar{ \beta}_{m}Z\eta^{T}\bigl(k-\tau_{m}(k)\bigr) \bar{A}_{2i}^{T}P\sum_{l=0}^{\ell} \bar{\alpha}_{l}\tilde{B}_{t}g_{v}\bigl(Z \eta(k-l)\bigr) \\ &{}+\sum_{m=1}^{h}\bar{ \beta}_{m}(k)Z\eta^{T}\bigl(k-\tau_{m}(k)\bigr) \bar{A}_{2i}^{T}P\bar{A}_{2s}\sum _{m=1}^{h}\bar{\beta}_{m}(k)Z\eta\bigl(k- \tau_{m}(k)\bigr) \\ &{}+\tilde{\omega}(k)^{T}\mathcal{D}_{1ij}^{T}P \mathcal{D}_{1st}\tilde{\omega}(k) +2\tilde{\omega}(k)^{T} \mathcal{D}_{1ij}^{T}P\bar{r}\mathcal{F}\bigl(\eta(k)\bigr) \\ &{}+2\tilde{\omega}(k)^{T}\mathcal{D}_{1ij}^{T}P \sum_{l=1}^{\ell}\bar{\alpha}_{l} \bar{B}_{t}Z\eta(k-l) \\ &{}+2\tilde{\omega}(k)^{T}\mathcal{D}_{1ij}^{T}P \sum_{l=0}^{\ell}\bar{\alpha}_{l} \tilde{B}_{j}g_{v}\bigl(Z\eta(k-l)\bigr) +\bar{r} \mathcal{F}^{T}\bigl(\eta(k)\bigr)P\bar{r}\mathcal{F}\bigl(\eta(k)\bigr) \\ &{}+2\bar{r}\mathcal{F}^{T}\bigl(\eta(k)\bigr)P\sum _{l=1}^{\ell}\bar{\alpha}_{l}\bar {B}_{t}Z\eta(k-l) +2\bar{r}\mathcal{F}^{T}\bigl(\eta(k) \bigr)P\sum_{l=0}^{\ell}\bar{ \alpha}_{l}\tilde{B}_{t}g_{v}\bigl(Z\eta(k-l) \bigr) \\ &{}+\tilde{r}(k)\mathcal{F}^{T}\bigl(\eta(k)\bigr)P\tilde{r}(k) \mathcal{F}\bigl(\eta(k)\bigr) +\sum_{l=1}^{\ell} \bar{\alpha}_{l}\eta^{T}(k-l)Z^{T} \bar{B}_{t}^{T}P\sum_{l=1}^{\ell} \bar{\alpha}_{l}\bar{B}_{t}Z\eta(k-l) \\ &{}+2\sum_{l=1}^{\ell}\bar{ \alpha}_{l}\eta^{T}(k-l)Z^{T}\bar{B}_{t}^{T}P \sum_{l=0}^{\ell}\bar{\alpha}_{l} \tilde{B}_{t}g_{v}\bigl(Z\eta(k-l)\bigr) \\ &{}+\sum_{l=0}^{\ell}\tilde{ \alpha}_{l}(k)\eta^{T}(k-l)Z^{T} \bar{B}_{t}^{T}P\sum_{l=0}^{\ell} \tilde{\alpha}_{l}(k)\bar{B}_{t}Z\eta(k-l) \\ &{}+2\sum_{l=0}^{\ell}\tilde{ \alpha}_{l}(k)\eta^{T}(k-l)Z^{T} \bar{B}_{t}^{T}P\sum_{l=0}^{\ell} \tilde{\alpha}_{l}(k)\tilde{B}_{t}g_{v}\bigl(Z \eta(k-l)\bigr) \\ &{}+\sum_{l=0}^{\ell}\tilde{ \alpha}_{l}(k)g_{v}^{T}\bigl(Z\eta(k-l)\bigr) \tilde{B}_{t}^{T}P\sum_{l=0}^{\ell} \tilde{\alpha}_{l}(k)\tilde{B}_{t}g_{v}\bigl(Z \eta(k-l)\bigr)\Biggr] -\eta(k)^{T}P\eta(k)\Biggr\} . \end{aligned}$$

Considering Lemma 2 and taking the elementary inequality $2ab\leq a^{2}+b^{2}$ into consideration, we obtain

20 $$\begin{aligned} \mathbb{E}\bigl\{ \Delta V_{1}(k)\bigr\} \leq{}& \mathbb{E}\Biggl\{ \sum_{i=1}^{n}\sum _{j=1}^{n}h_{i}h_{j}\Biggl[ \eta(k)^{T}\bigl(\bar{A}_{1ij}^{T}P \bar{A}_{1ij}-P\bigr)\eta(k) \\ &{}+2\eta(k)^{T}\bar{A}_{1ij}^{T}P \bar{A}_{2i}\sum_{m=1}^{h}\bar{ \beta}_{m}Z\eta\bigl(k-\tau_{m}(k)\bigr) +2 \eta(k)^{T}\bar{A}_{1ij}^{T}P\mathcal{D}_{1ij} \tilde{\omega}(k) \\ &{}+2\eta(k)^{T}\bar{A}_{1ij}^{T}P\bar{r} \mathcal{F}\bigl(\eta(k)\bigr) +2\eta(k)^{T}\bar{A}_{1ij}^{T}P \sum_{l=1}^{\ell}\bar{\alpha}_{l} \bar{B}_{j}Z\eta(k-l) \\ &{}+2\eta(k)^{T}\bar{A}_{1ij}^{T}P\sum _{l=0}^{\ell}\bar{\alpha}_{l}\tilde {B}_{j}g_{v}\bigl(Z\eta(k-l)\bigr) \\ &{}+\Biggl(\sum_{m=1}^{h}\bar{ \beta}_{m}Z\eta^{T}\bigl(k-\tau_{m}(k)\bigr) \bar{A}_{2i}\Biggr)^{T}P\bar{A}_{2i}\sum _{m=1}^{h}\bar{\beta}_{m}Z\eta\bigl(k- \tau_{m}(k)\bigr) \\ &{}+2\Biggl(\sum_{m=1}^{h}\bar{ \beta}_{m}Z\eta^{T}\bigl(k-\tau_{m}(k)\bigr)\bar {A}_{2i}\Biggr)^{T}P\mathcal{D}_{1ij}\tilde{ \omega}(k) \\ &{}+2\Biggl(\sum_{m=1}^{h}\bar{ \beta}_{m}Z\eta^{T}\bigl(k-\tau_{m}(k)\bigr) \bar{A}_{2i}\Biggr)^{T}P\bar{r}\mathcal{F}\bigl(\eta(k)\bigr) \\ &{}+2\Biggl(\sum_{m=1}^{h}\bar{ \beta}_{m}Z\eta^{T}\bigl(k-\tau_{m}(k)\bigr) \bar{A}_{2i}\Biggr)^{T}P\sum_{l=1}^{\ell} \bar{\alpha}_{l}\bar{B}_{j}Z\eta(k-l) \\ &{}+2\Biggl(\sum_{m=1}^{h}\bar{ \beta}_{m}Z\eta^{T}\bigl(k-\tau_{m}(k)\bigr) \bar{A}_{2i}\Biggr)^{T}P\sum_{l=0}^{\ell} \bar{\alpha}_{l}\tilde{B}_{j}g_{v}\bigl(Z \eta(k-l)\bigr) \\ &{}+\Biggl(\sum_{m=1}^{h}\bar{ \beta}_{m}(k)Z\eta^{T}\bigl(k-\tau_{m}(k)\bigr) \bar{A}_{2i}\Biggr)^{T}P\bar{A}_{2s}\sum _{m=1}^{h}\bar{\beta}_{m}(k)Z\eta\bigl(k- \tau_{m}(k)\bigr) \\ &{}+\tilde{\omega}(k)^{T}\mathcal{D}_{1ij}^{T}P \mathcal{D}_{1ij}\tilde{\omega}(k) +2\tilde{\omega}(k)^{T} \mathcal{D}_{1ij}^{T}P\bar{r}\mathcal{F}\bigl(\eta(k)\bigr) \\ &{}+2\tilde{\omega}(k)^{T}\mathcal{D}_{1ij}^{T}P \sum_{l=1}^{\ell}\bar{\alpha}_{l} \bar{B}_{j}Z\eta(k-l)+2\tilde{\omega}(k)^{T}\mathcal{D}_{1ij}^{T}P \sum_{l=0}^{\ell}\bar{\alpha}_{l} \tilde{B}_{j}g_{v}\bigl(Z\eta(k-l)\bigr) \\ &{} +\bar{r} \mathcal{F}^{T}\bigl(\eta(k)\bigr)P\bar{r}\mathcal{F}\bigl(\eta(k)\bigr) +2\bar{r}\mathcal{F}^{T}\bigl(\eta(k)\bigr)P\sum _{l=1}^{\ell}\bar{\alpha}_{l}\bar {B}_{j}Z\eta(k-l) \\ &{} +2\bar{r}\mathcal{F}^{T}\bigl(\eta(k) \bigr)P\sum_{l=0}^{\ell}\bar{ \alpha}_{l}\tilde{B}_{j}g_{v}\bigl(Z\eta(k-l) \bigr)+\tilde{r}^{2}\mathcal{F}^{T}\bigl(\eta(k)\bigr)P \mathcal{F}\bigl(\eta(k)\bigr) \\ &{} +\sum_{l=1}^{\ell} \bar{\alpha}_{l}\eta^{T}(k-l)Z^{T} \bar{B}_{j}^{T}P\sum_{l=1}^{\ell} \bar{\alpha}_{l}\bar{B}_{j}Z\eta(k-l)+2\sum_{l=1}^{\ell}\bar{ \alpha}_{l}\eta^{T}(k-l)Z^{T}\bar{B}_{j}^{T} \\ &{}\times P \sum_{l=0}^{\ell}\bar{\alpha}_{l} \tilde{B}_{j}g_{v}\bigl(Z\eta(k-l)\bigr) +\sum_{l=0}^{\ell}\tilde{ \alpha}_{l}^{2}\eta^{T}(k-l)Z^{T} \bar{B}_{j}^{T}P\bar{B}_{j}Z\eta(k-l) \\ &{}+2\sum_{l=0}^{\ell}\tilde{ \alpha}_{l}^{2}\eta^{T}(k-l)Z^{T} \bar{B}_{j}^{T}P\tilde{B}_{j}g_{v} \bigl(Z\eta(k-l)\bigr) \\ &{}+\sum_{l=0}^{\ell}\tilde{ \alpha}_{l}^{2}g_{v}^{T}\bigl(Z\eta(k-l) \bigr)\tilde{B}_{j}^{T}P\tilde{B}_{j}g_{v} \bigl(Z\eta(k-l)\bigr)\Biggr]\Biggr\} . \end{aligned}$$

Also, it can be seen that

21 $$\begin{aligned} \mathbb{E}\bigl\{ \Delta V_{2}(k)\bigr\} ={}&E\bigl\{ V_{2}(k+1)-V_{2}(k)\bigr\} \\ ={}&\mathbb{E}\Biggl\{ \sum_{m=1}^{h}\sum _{i=k+1-\tau_{m}(k)}^{k}\eta^{T}(i)Z^{T}Q_{m}Z \eta(i)- \sum_{m=1}^{h}\sum _{i=k-\tau_{m}(k)}^{k-1}\eta^{T}(i)Z^{T}Q_{m}Z \eta(i)\Biggr\} \\ \leq{}&\mathbb{E}\Biggl\{ \eta^{T}(k)Z^{T}Q_{m}Z \eta(k)-\eta^{T}\bigl(k-\tau_{m}(k)\bigr)Z^{T}Q_{m}Z \eta\bigl(k-\tau_{m}(k)\bigr) \\ &{}+\sum_{i=k-\bar{d}_{m}+1}^{k-\underline{d}_{m}}\eta^{T}(i)Z^{T}Q_{m}Z \eta(i)\Biggr\} , \end{aligned}$$

22 $$\begin{aligned} \mathbb{E}\bigl\{ \Delta V_{3}(k)\bigr\} ={}&E\bigl\{ V_{3}(k+1)-V_{3}(k)\bigr\} \\ ={}&\mathbb{E}\Biggl\{ \sum_{m=1}^{h}\sum _{n=-\bar{d}_{m}+1}^{-\underline{d}_{m}}\sum_{i=k+n+1}^{k} \eta^{T}(i)Z^{T}Q_{m}Z\eta(i) \\ &{}-\sum_{m=1}^{h}\sum _{n=-\bar{d}_{m}+1}^{-\underline{d}_{m}}\sum_{i=k+n}^{k-1} \eta^{T}(i)Z^{T}Q_{m}Z\eta(i)\Biggr\} \\ \leq{}&\mathbb{E}\Biggl\{ \sum_{m=1}^{h} \Biggl[(\bar{d}_{m}-\underline{d}_{m})\eta ^{T}(k)Z^{T}Q_{m}Z\eta(k) \\ &{}- \sum _{i=k-\bar{d}_{m}+1}^{k-\underline{d}_{m}}\eta^{T}(i)Z^{T}Q_{m}Z \eta(i)\Biggr]\Biggr\} , \end{aligned}$$

and

23 $$ \begin{aligned}[b] \mathbb{E}\bigl\{ \Delta V_{4}(k)\bigr\} &=E\bigl\{ V_{4}(k+1)-V_{4}(k)\bigr\} \\ &=\mathbb{E}\Biggl\{ \sum_{l=1}^{\ell}\sum _{i=k+1-l}^{k}\eta^{T}(i)Z^{T} \mathcal{R}_{l}Z\eta(i) -\sum_{l=1}^{\ell} \sum_{i=k-l}^{k-1}\eta^{T}(i)Z^{T} \mathcal{R}_{l}Z\eta(i)\Biggr\} \\ &=\mathbb{E}\Biggl\{ \sum_{l=1}^{\ell}\bigl( \eta^{T}(k)Z^{T}\mathcal{R}_{l}Z\eta(k)- \eta^{T}(k-l)Z^{T}\mathcal{R}_{l}Z\eta(k-l)\bigr) \Biggr\} . \end{aligned} $$

For notational convenience, we have

$$\begin{aligned}& \eta(k-\tau)=\bigl[\eta\bigl(k-\tau_{1}(k)\bigr)^{T}Z^{T}, \eta\bigl(k-\tau_{2}(k)\bigr)^{T}Z^{T},\ldots, \eta\bigl(k-\tau_{m}(k)\bigr)^{T}Z^{T} \bigr]^{T}, \\& \eta(k)^{\ell}=\bigl[\eta(k)^{T}Z^{T}, \eta(k-1)^{T}Z^{T},\ldots,\eta(k-\ell)^{T}Z^{T} \bigr]^{T}, \\& \mathcal{G}(k)^{\ell}=\bigl[g_{v}^{T}\bigl(Z \eta(k)\bigr),g_{v}^{T}\bigl(Z\eta(k-1)\bigr), \ldots,g_{v}^{T}\bigl(Z\eta(k-\ell)\bigr) \bigr]^{T}, \\& \bar{\eta}(k)=\bigl[\eta(k)^{T},\eta(k-\tau)^{T}, \eta(k)^{\ell T},\mathcal{F}^{T}\bigl(\eta(k)\bigr), \mathcal{G}(k)^{\ell T}\bigr]^{T}, \\& \tilde{\eta}(k)=\bigl[\eta(k)^{T},\eta(k-\tau)^{T}, \eta(k)^{\ell T},\mathcal{F}^{T}\bigl(\eta(k)\bigr), \mathcal{G}(k)^{\ell T},\tilde{\omega}^{T}(k)\bigr]^{T}. \end{aligned}$$

In the first place, we will prove the exponential stability of the system (12) with $\tilde{\omega}(k)=0$, considering (5), (13), Lemma 2, and Lemma 3, we can get

$$\begin{aligned} &\mathbb{E}\bigl\{ \Delta V(k)\bigr\} \\ &\quad\leq\mathbb{E}\Biggl\{ \sum_{i=1}^{4} \Delta V_{i}(k)-\varphi\bigl[\mathcal{F}^{T}\bigl(\eta(k) \bigr)\mathcal{F}\bigl(\eta(k)\bigr)- \lambda\eta^{T}(k)\mathcal {G}^{T}\mathcal{G}\eta(k)\bigr] \\ &\qquad{}-2\psi\bigl[\mathcal{G}(k)^{\ell T}\mathcal{G}(k)^{\ell}- \mathcal{G}(k)^{\ell T}\bigl(I_{\ell+1}\otimes M^{T}M \bigr)\eta_{k}^{\ell}\bigr]\Biggr\} \\ &\quad\leq\mathbb{E}\Biggl\{ \sum_{i=1}^{4} \Delta V_{i}(k)-\varphi\bigl[\mathcal{F}^{T}\bigl(\eta(k) \bigr)\mathcal{F}\bigl(\eta(k)\bigr)- \lambda\eta^{T}(k)\mathcal {G}^{T}\mathcal{G}\eta(k)\bigr] \\ &\qquad{}-\psi\mathcal{G}(k)^{\ell T}\mathcal{G}(k)^{\ell}+\psi \eta(k)^{\ell T}\bigl(I_{\ell+1}\otimes M^{T}M\bigr) \eta(k)^{\ell}\Biggr\} \\ &\quad\leq\mathbb{E}\Biggl\{ \sum_{i=1}^{h} \sum_{j=1}^{h}h_{i}h_{j} \bar{\eta}(k)^{T}\Gamma_{1ij}\bar{\eta}(k)\Biggr\} , \end{aligned}$$

where

24 $$\begin{aligned}& {{\Gamma_{1ij}= \begin{bmatrix} \Gamma_{11ij}+\varphi\lambda\mathcal{G}^{T}\mathcal{G} & \Gamma_{12ij} & \Gamma_{13ij} & \Gamma_{14ij} & \Gamma_{15ij}\\ {*} & \Gamma_{22ii}+I & \Gamma_{23ij} & \Gamma_{24ii} &\Gamma_{25ij}\\ {*} & * & \Gamma_{33jj}+\psi I_{\ell+1}\otimes(M^{T}M) & \Gamma_{34jj} & \Gamma_{35jj}\\ {*} & * & * & \Gamma_{44}-\varphi I & \Gamma_{45jj}\\ {*} & * & * & * & \Gamma_{55jj}-\psi I \end{bmatrix} ,}} \\& \Gamma_{11ij}=\bar{A}_{1ij}^{T}P \bar{A}_{1ij}+\tilde{\alpha}_{0}^{2}Z^{T} \bar{A}_{2i}^{T}P\bar{A}_{2i} Z+\sum _{l=1}^{\ell}Z^{T}R_{l}Z^{T}+Z^{T}Z +\sum_{m=1}^{h}(\bar{d}_{m}- \underline{d}_{m}+1)Z^{T}Q_{m} Z-P, \\& \Gamma_{12ij}=\bigl(\bar{\beta}_{1}\bar{A}_{1ij}^{T}P \bar{A}_{2i},\ldots,\bar{\beta}_{h}\bar{A}_{1ij}^{T}P \bar{A}_{2i}\bigr), \\& \Gamma_{13ij}=\bigl(0,\bar{\alpha}_{1}\bar{A}_{1ij}^{T}P \bar{B}_{j},\ldots,\bar{\alpha}_{\ell}\bar{A}_{1ij}^{T}P \bar{B}_{j}\bigr), \\& \Gamma_{14ij}=\bar{r}\bar{A}_{1ij}^{T}P, \\& \Gamma_{15ij}=\bigl(\bar{\alpha}_{0}\bar{A}_{1ij}^{T}P \tilde{B}_{j},\ldots,\bar{\alpha}_{\ell}\bar{A}_{1ij}^{T}P \tilde{B}_{j}\bigr), \\& \Gamma_{22ii}=(\bar{\Theta}_{1}\otimes\bar{A}_{2i})^{T}P( \bar{\Theta}_{1}\otimes\bar{A}_{2i})+ (\tilde{ \Theta}_{1}\otimes\bar{A}_{2i})^{T} \mathcal{P}_{h}(\tilde{\Theta}_{1}\otimes \bar{A}_{2i})-\mathcal{Q}_{h}, \\& \Gamma_{23ij}=(\bar{\Theta}_{1}\otimes I)^{T} \bigl(0,\bar{\alpha}_{1}\bar{A}_{2i}^{T}P \bar{B}_{j},\ldots,\bar{\alpha}_{\ell}\bar{A}_{2i}^{T}P \bar{B}_{j}\bigr), \\& \Gamma_{24ii}=\bigl(\bar{\beta}_{1}\bar{\delta} \bar{A}_{2i}^{T}P, \ldots,\bar{\beta}_{h}\bar{ \delta}\bar{A}_{2i}^{T}P\bigr), \\& \Gamma_{25ij}=(\bar{\Theta}_{1}\otimes I)^{T} \bigl(0,\bar{\alpha}_{1}\bar{A}_{2i}^{T}P \tilde{B}_{j},\ldots,\bar{\alpha}_{\ell}\bar {A}_{2i}^{T}P\tilde{B}_{j}\bigr), \\& \Gamma_{33jj}=(\bar{\Theta}_{2}\otimes\bar{B}_{j})^{T}P( \bar{\Theta}_{2}\otimes\bar{B}_{j})+ (\tilde{ \Theta}_{3}\otimes\bar{B}_{j})^{T} \mathcal{P}_{\ell+1}(\tilde{\Theta}_{3}\otimes \bar{B}_{j})-\mathcal{R}_{\ell}, \\& \Gamma_{34jj}=\bigl(0,\bar{\alpha}_{1}\bar{\delta} \bar{B}_{j}^{T}P,\ldots,\bar{\alpha}_{\ell}\bar{ \delta}\bar{B}_{j}^{T}P\bigr), \\& \Gamma_{35jj}=(\bar{\Theta}_{2}\otimes\bar{B}_{j})^{T}P( \bar{\Theta}_{3}\otimes\tilde{B}_{j})+ (\tilde{ \Theta}_{3}\otimes\bar{B}_{j})^{T} \mathcal{P}_{\ell+1}(\tilde{\Theta}_{3}\otimes \tilde{B}_{j}), \\& \Gamma_{44}=\bar{r}^{2}P+\tilde{r}^{2}P,\qquad \Gamma_{45jj}=(\bar{\alpha}_{0}\bar{\delta}P \tilde{B}_{j},\bar{\alpha}_{1}\bar{\delta}P \tilde{B}_{j},\ldots,\bar{\alpha}_{\ell}\bar{\delta}P\tilde {B}_{j}), \\& \Gamma_{55jj}=(\bar{\Theta}_{3}\otimes\tilde{B}_{j})^{T}P( \bar{\Theta}_{3}\otimes\tilde{B}_{j})+(\tilde{ \Theta}_{3}\otimes\tilde{B}_{j})^{T} \mathcal{P}_{\ell+1}(\tilde{\Theta}_{3}\otimes\tilde {B}_{j}). \end{aligned}$$

By utilizing Lemma 1, we know that (16) and (17) implies $\mathbb{E}\{\Delta V(k)\}\leq0$ is true. Moreover, we can draw the conclusion that the nominal control system (10) with $\tilde{\omega}(k)=0$ is exponentially mean-square stable as can be seen in the same way as in [33].

Now let us dispose of the $H_{\infty}$ performance for the system (12). For this purpose, we establish a cost function

25 $$ \mathbf{J}(n)=\mathbb{E}\sum_{k=0}^{n} \bigl[z^{T}(k)z(k)-\gamma^{2}\tilde{\omega}^{T}(k) \tilde{\omega}(k)\bigr]. $$

There is no doubt that we can show $\mathbf{J}(n)<0$ under the zero-initial condition, which is our purpose.

Along the trajectory of the fuzzy discrete-time system (12) and taking (25) into consideration, we have

26 $$\begin{aligned} \mathbf{J}(n)&=\mathbb{E}\Biggl\{ \sum _{k=0}^{n}\bigl[z^{T}(k)z(k)- \gamma^{2}\tilde{\omega}^{T}(k)\tilde{\omega} +\Delta V(k) \bigr]-V(n+1)\Biggr\} \\ &\leq\mathbb{E}\Biggl\{ \sum_{k=0}^{n} \bigl[z^{T}(k)z(k)-\gamma^{2}\tilde{\omega}^{T}(k) \tilde{\omega}(k) +\Delta V(k)\bigr]\Biggr\} \\ &\leq\sum_{k=0}^{n}\mathbb{E}\bigl\{ \Delta V(k)+\bigl(\mathcal{E}_{i}\eta(k)+\mathcal{D}_{2j}\tilde{ \omega}(k)\bigr)^{T} \bigl(\mathcal{E}_{i}\eta(k)+ \mathcal{D}_{2j}\tilde{\omega}(k)\bigr) -\gamma^{2}\tilde{ \omega}^{T}(k)\tilde{\omega}(k)\bigr\} \\ &\leq\sum_{i=1}^{h}\sum _{j=1}^{h}h_{i}h_{j}\bigl[ \tilde{\eta}(k)^{T}\Gamma_{2ij}\tilde{\eta}(k)\bigr], \end{aligned}$$

where

$$\begin{aligned}& \Gamma_{2ij}=\left [ \textstyle\begin{array}{@{}c@{\quad}c@{\quad}c@{}} \Gamma_{11ij}+\varphi\lambda\mathcal{G}^{T}\mathcal{G} +\mathcal{E}_{i}^{T}\mathcal{E}_{i} & \Gamma_{12ij} & \Gamma_{13ij}\\ {*} & \Gamma_{22ii}+I & \Gamma_{23ij}\\ {*} & * & \Gamma_{33jj}+\psi I_{\ell+1}\otimes(M^{T}M)\\ {*} & * & * \\ {*} & * & * \\ {*} & * & * \end{array}\displaystyle \right . \\& \left . \hphantom{\Gamma_{2ij}=[\Gamma_{2ij}=[\Gamma_{2ij}=[\Gamma_{2ij}=[}{} \textstyle\begin{array}{@{}c@{\quad}c@{\quad}c@{}} \Gamma_{14ij} & \Gamma_{15ij} & \Gamma_{16ij}\\ \Gamma_{24ii} & \Gamma_{25ij} & \Gamma_{26ij}\\ \Gamma_{34jj} & \Gamma_{35jj} & \Gamma_{36ij}\\ \Gamma_{44}-\varphi I & \Gamma_{45jj} & \Gamma_{46ij}\\ {*} & \Gamma_{55jj}-\psi I & \Gamma_{56ij}\\ {*} & * & \Gamma_{66ij}+\mathcal{D}_{2j}^{T}\mathcal{D}_{2j}-\gamma^{2}I \end{array}\displaystyle \right ], \\& \Gamma_{16ij}=\bar{A}_{1ij}^{T}P \mathcal{D}_{1ij},\qquad\Gamma_{26ij}=\bigl(\bar{ \beta}_{1}\mathcal{D}_{1ij}^{T}P \bar{A}_{2i},\ldots,\bar{\beta}_{h}\mathcal{D}_{1ij}^{T}P \bar{A}_{2i}\bigr)^{T}, \\& \Gamma_{36ij}=\bigl(0,\bar{\alpha}_{1}\mathcal{D}_{1ij}^{T}P \bar{B}_{j},\ldots,\bar{\alpha}_{\ell}\mathcal{D}_{1ij}^{T}P \bar{B}_{j}\bigr)^{T},\qquad\Gamma_{46ij}=\bar{r}P \mathcal{D}_{1ij}, \\& \Gamma_{56ij}=\bigl(\bar{\alpha}_{0}\mathcal{D}_{1ij}^{T}P \tilde{B}_{j},\ldots,\bar{\alpha}_{\ell}\mathcal{D}_{1ij}^{T}P \tilde{B}_{j}\bigr),\qquad\Gamma_{66ij}=\mathcal{D}_{1ij}^{T}P \mathcal{D}_{1ij}. \end{aligned}$$

By using the Schur complement lemma, the conclusion can be drawn from (16) and (17) that $\mathbf{J}(n)<0$. Letting $n\rightarrow\infty$, it follows from the above inequality that

$$\sum_{k}^{\infty}\mathbb{E}\bigl\{ \bigl\| z(k) \bigr\| ^{2}\bigr\} \leq\gamma^{2}\sum_{k=0}^{\infty} \bigl\| \tilde{\omega}(k)\bigr\| ^{2}, $$

which completes the proof of Theorem 1. □

Through the above-mentioned analysis results for the control problem, we will deal with the problem of designing the desired $H_{\infty}$ fuzzy output-feedback controller in the following theorem.

### Theorem 2

*Let the* $H_{\infty}$ *disturbance attenuation level* $\gamma>0$ *be given*. *A desired controller of the form* (11) *exists if there exist matrices* $S>0$, $T>0$, $Q_{m}>0$ ($m=1,\ldots,h$), $R_{l}>0$ ($l=0,1,\ldots ,\ell$), *matrices* $K_{1j}$, $K_{2j}$, $K_{3j}$ ($j=1,\ldots,r$), *and a positive scalar* $\varphi>0$ *and* $\psi>0$ *satisfying*

27 $$\begin{aligned}& \Upsilon_{ii}^{3}< 0, \end{aligned}$$

28 $$\begin{aligned}& 2\bigl(\Upsilon_{ij}^{3}+\Upsilon_{ji}^{3} \bigr)< 0, \end{aligned}$$

*where* $1\leq i < j \leq r$ ($i,j\in R$)

$$\begin{aligned}& \Upsilon_{ij}^{3}= \begin{bmatrix} \varLambda _{11}^{3} & \varOmega _{12ij}^{3} & 0\\ {*} & \varLambda _{22}^{3} & \varOmega _{23ii}^{3}\\ {*} & * & \varLambda _{33}^{3} \end{bmatrix} , \\& \varLambda _{11}^{3}=\operatorname{diag}\{\hat{P},\hat{ \mathcal{P}}_{\ell+1},-I,-\varphi I\}, \\& \varLambda _{22}^{3}=\operatorname{diag}\bigl\{ -\tilde{P},- \mathcal{Q}_{h},-\mathrm{R}_{\ell },-\varphi I,-\psi I,- \gamma^{2}I\bigr\} , \\& \Gamma_{33}^{1}=\operatorname{diag}\{-\tilde{ \mathcal{P}}_{h},-\tilde{P},-\bar{\mathcal{Q}}_{m},- \mathcal{R}_{\ell}, -\psi I_{\ell+1}\}, \\& \varOmega _{12ij}^{1}= \begin{bmatrix} \bar{\mathcal{A}}_{1ij} & \bar{\Theta}_{1}\otimes\bar{\mathcal{A}}_{2i} & \bar{\Theta}_{2}\otimes\bar{\mathcal{B}} & \bar{r}\bar{W}_{2} & \bar {\Theta}_{3}\otimes\tilde{\mathcal{B}} & \bar{\mathcal{D}}_{1ij}\\ 0 & 0 & \tilde{\Theta}_{3}\otimes\bar{\mathcal{B}} & 0 & \tilde{\Theta }_{3}\otimes\tilde{\mathcal{B}} & 0\\ \bar{\mathcal{E}}_{i} & 0 & 0 & 0 & 0 & \mathcal{D}_{2j}\\ \varphi\sqrt{\lambda}\bar{\mathcal{G}} & 0 & 0 & 0 & 0 & 0 \end{bmatrix} , \\& \varOmega _{23ii}^{1}= \begin{bmatrix} 0 & 0 & Z^{T}\bar{\mathcal{Q}}_{m} & Z^{T}\otimes\bar{\mathcal{R}}_{\ell} & 0\\ \tilde{\Theta}_{1}\otimes\tilde{\mathcal{A}}_{2i}^{T} & 0 & 0 & 0 & 0\\ 0 & 0 & 0 & 0 & \psi\mathcal{M}^{T}\\ 0 & \tilde{r}\mathcal{T} & 0 & 0 & 0\\ 0 & 0 & 0 & 0 & 0\\ 0 & 0 & 0 & 0 & 0 \end{bmatrix} , \\& \hat{P}= \begin{bmatrix} \bar{T} & -I\\ {*} & -S \end{bmatrix} ,\qquad\tilde{P}= \begin{bmatrix} T& T\\ * & S \end{bmatrix} , \qquad\bar{W}_{2}= \begin{bmatrix} I& 0\\ S & 0 \end{bmatrix} , \\& \hat{\mathcal{P}}_{s}=\operatorname{diag}\{\underbrace{\hat{P}, \ldots,\hat{P}}_{s}\},\qquad\tilde{\mathcal{P}}_{s}= \operatorname{diag}\{\underbrace{\tilde{P},\ldots,\tilde{P}}_{s}\}, \qquad\mathcal{T}=\operatorname{diag}\{T,I\}, \\& \bar{\mathcal{E}}_{i}= \begin{bmatrix} E \\ E \end{bmatrix} ,\qquad\bar{ \mathcal{G}}= \begin{bmatrix} G&0 \\ 0&G \end{bmatrix} ,\qquad\bar{T}=-H-H^{T}+H^{T}TH, \\& \bar{\mathcal{R}_{l}}=(I,R_{1},\ldots,R_{\ell}), \\& \bar{\mathcal{A}}_{1ij}= \begin{bmatrix} A_{1ij}+B_{i} K_{3j} & A_{1ij}\\ SA_{1ij}+\bar{\alpha}_{0}K_{2j}M_{1}+K_{1j} & SA_{1ij}+\bar{\alpha}_{0}K_{2j}M_{1} \end{bmatrix} ,\qquad \bar{A}_{2i}= \begin{bmatrix} A_{2i}\\ SA_{2i} \end{bmatrix} , \\& \tilde{A}_{2i}= \begin{bmatrix} TA_{2i}\\ SA_{2i} \end{bmatrix} ,\qquad\bar{\mathcal{B}}= \begin{bmatrix} 0\\ K_{2j}M_{1} \end{bmatrix} ,\qquad\tilde{\mathcal{B}}= \begin{bmatrix} 0\\ K_{2j} \end{bmatrix} ,\qquad \bar{\mathcal{D}}_{1ij}= \begin{bmatrix} D_{1ij} & 0\\ SD_{1ij} & K_{2j} \end{bmatrix} , \end{aligned}$$

*the controller parameters in the form of* (8) *are given in the following*:

29 $$\begin{aligned} &A_{cj}=X_{12}\bigl\{ K_{1j}[T-S]^{-1}X_{12}-S B_{i} C_{cj}\bigr\} , \\ &B_{cj}=X_{12}^{-1}K_{2j},\qquad C_{cj}=K_{3j}[T-S]^{-1}X_{12}, \end{aligned}$$

*where the matrix* $X_{12}$ *derives from the factorization* $I-ST^{-1}=X_{12}Y_{12}^{T}<0$, *and then the fuzzy discrete*-*time closed*-*loop system* (12) *is exponentially mean*-*square stable and the controlled output* $z_{k}$ *satisfies* (15).

### Proof

For the purpose of design desired controller parameters $A_{cj}, B_{cj}$, and $C_{cj}$ from Theorem 1, we partition *P* and $P^{-1}$ as

$$(*)\quad P= \begin{bmatrix} S & X_{12} \\ X_{12}^{T} & X_{22} \end{bmatrix} ,\qquad P^{-1}= \begin{bmatrix} T^{-1} & Y_{12} \\ Y_{12}^{T} & Y_{22} \end{bmatrix} , $$

where the partitioning of *P* and $P^{-1}$ are appropriately dimensioned to be determined by $\bar{A}_{1ij}, \bar{A}_{2i}, \mathcal {D}_{1ij}$, and $\bar{B}_{j}$ in (12).

Define

$$W_{1}= \begin{bmatrix} T^{-1} & I \\ Y_{12}^{T} & 0 \end{bmatrix} ,\qquad W_{2}= \begin{bmatrix} I & S \\ 0 & X_{22}^{T} \end{bmatrix} , $$

and then we have $PW_{1}=W_{2}$ and $W_{1}^{T}PW_{1}=W_{1}^{T}W_{2}$. Now we define the controller parameters from (29) as follows:

30 $$\begin{aligned} &K_{1j}=[SB_{i}C_{cj}+X_{12}A_{cj}]Y_{12}^{T}T, \\ &K_{2j}=X_{12}B_{cj}, \\ &K_{3j}=C_{cj}Y_{12}^{T}T. \end{aligned}$$

By applying the congruence transformation

$$\operatorname{diag}\{W_{1},\underbrace{W_{1}, \ldots,W_{1}}_{\ell+1}, I,I,W_{1},I_{h},I_{\ell+1},I,I,I, \underbrace{W_{1},\ldots,W_{1}}_{h},W_{1},I,I_{\ell+1},I_{\ell+1} \} $$

to (16) and (17), we can have

31 $$\begin{aligned}& \begin{bmatrix} \varLambda _{11}^{2} & \varOmega _{12ij}^{2} & 0\\ * & \varLambda _{22}^{2} & \varOmega _{23ii}^{2}\\ * & * & \varLambda _{33}^{2} \end{bmatrix} < 0, \\& \varLambda _{11}^{2}=\operatorname{diag}\{-\bar{P},-\bar{ \mathcal{P}}_{\ell+1},-I,-\varphi I\}, \\& \varLambda _{22}^{2}=\operatorname{diag}\bigl\{ -\bar{P},- \mathcal{Q}_{h},-\mathrm{R}_{\ell },-\varphi I,-\psi I,- \gamma^{2}I\bigr\} , \\& \Gamma_{33}^{2}=\operatorname{diag}\bigl\{ -\bar{ \mathcal{P}}_{h},-\bar{P},-\bar{\mathcal{Q}}_{m}^{-1},- \mathcal{R}_{\ell}^{-1}, -\psi I_{\ell+1}\bigr\} , \\& \varOmega _{12ij}^{2}= \begin{bmatrix} \tilde{\mathcal{A}}_{1ij} & \bar{\Theta}_{1}\otimes\bar{\mathcal{A}}_{2i} & \bar{\Theta}_{2}\otimes\bar{\mathcal{B}} & \bar{r}\bar{W}_{2} & \bar {\Theta}_{3}\otimes\tilde{\mathcal{B}} & \bar{\mathcal{D}}_{1ij}\\ 0 & 0 & \tilde{\Theta}_{3}\otimes\bar{\mathcal{B}} & 0 & \tilde{\Theta }_{3}\otimes\tilde{\mathcal{B}} & 0\\ \tilde{\mathcal{E}}_{i} & 0 & 0 & 0 & 0 & \mathcal{D}_{2j}\\ \varphi\sqrt{\lambda}\tilde{\mathcal{G}} & 0 & 0 & 0 & 0 & 0 \end{bmatrix} , \\& \varOmega _{23ii}^{2}= \begin{bmatrix} 0 & 0 & Z^{T}T_{-1} & Z^{T}T_{-1}\otimes\bar{I}_{\ell} & 0\\ \tilde{\Theta}_{1}\otimes\bar{\mathcal{A}}_{2i}^{T} & 0 & 0 & 0 & 0\\ 0 & 0 & 0 & 0 & \psi\mathcal{M}^{T}\\ 0 & \tilde{r}W_{2} & 0 & 0 & 0\\ 0 & 0 & 0 & 0 & 0\\ 0 & 0 & 0 & 0 & 0 \end{bmatrix} , \\& \bar{P}= \begin{bmatrix} T^{-1} & I\\ * & S \end{bmatrix} ,\qquad\bar{\mathcal{P}}_{s}= \operatorname{diag}\{\underbrace{\bar{P},\ldots,\bar{P}}_{s}\}, \\& \bar{\mathcal{E}}_{i}= [ \textstyle\begin{array}{@{}c@{\quad}c@{}} E_{i}T^{-1} & E_{i} \end{array}\displaystyle ],\qquad \bar{\mathcal{G}}= [ \textstyle\begin{array}{@{}c@{\quad}c@{}} GT^{-1} & G \end{array}\displaystyle ],\qquad \bar{I}_{l}=[\underbrace{I,\ldots,I}_{\ell+1}], \\& \tilde{\mathcal{A}}_{1ij}= \begin{bmatrix} (A_{1ij}+B_{i} K_{3j})T^{-1} & A_{1ij}\\ (SA_{1ij}+\bar{\alpha}_{0}K_{2j}M_{1}+K_{1j})T^{-1} & SA_{1ij}+\bar{\alpha }_{0}K_{2j}M_{1} \end{bmatrix} . \end{aligned}$$

On the other hand, it follows from $[T^{-1}-H]^{T}T[T^{-1}-H]\geq0$ that

32 $$ -T^{-1}\leq-H-H^{T}+H^{T}TH. $$

Furthermore, again applying the congruence transformation

$$\operatorname{diag}\{I,I_{\ell+1},I,I,\mathcal{T},I_{h},I_{\ell+1},I,I,I, \underbrace{\mathcal{T},\ldots,\mathcal{T}}_{h},\mathcal{T},\bar{ \mathcal{Q}}_{m},I_{\ell+1},\mathcal{R}_{\ell+1}\} $$

to (30), we have

33 $$ \begin{bmatrix} \varLambda _{11}^{2} & \varOmega _{12ij}^{3} & 0\\ * & \varLambda _{22}^{3} & \varOmega _{23ii}^{3}\\ * & * & \varLambda _{33}^{3} \end{bmatrix} < 0, $$

where $\mathcal{T}=\operatorname{diag}\{T,I\}$. By combination (31) and (32), if (27) and (28) are satisfied, the inequality (33) holds. Therefore the sufficient condition (16) and (17) of Theorem 1 is effective.

Next, let us calculate the desired controller parameters. We can obtain from $PP^{-1}=I$

34 $$ I-ST^{-1}=X_{12}Y_{12}. $$

By $P>0, T>0, S>0, W_{1}^{T}PW_{1}=W_{1}^{T}W_{2}= \bigl[ {\scriptsize\begin{matrix}{} T^{-1} & I \cr I & S \end{matrix}} \bigr]$, and if (27) and (28) are feasible, we can infer $I-ST^{-1}=X_{12}Y_{12}<0$. So $I-ST^{-1}$ is nonsingular. Hence one can always find square and nonsingular $X_{12}$ and $Y_{12}$ satisfying (34) [37]. In this case, we can obtain $A_{cj}, B_{cj}$, and $C_{cj}$ via solving (30). Now it can be concluded from Theorem 2 that the fuzzy closed-loop system (12) is exponentially mean-square stable and the controlled output $z_{(}k)$ satisfies (15) with the controller parameters given by (29). □

### Remark 4

The model considered in this paper is more general than some existing ones [22, 23, 25]. For example, when the model does not take into consideration the randomly occurring interval delay and randomly occurring nonlinearities, it reduces to the model in [25]. The results derived in this paper also contain the two theorems in [25] as special cases. Moreover, randomly occurring nonlinearities have not been considered in [22], and sector nonlinearities have not been studied in [23].

### Remark 5

The design of controller directly affects the stability and $H_{\infty}$ performance of the discrete-time closed-loop system. Compared with [7, 20, 21], it should be pointed out that the fuzzy controller designing arithmetic in Theorem 2 has more generality than the usual controller, that is to say, the controller designing arithmetic in [20, 21] cannot be available for the design of fuzzy $H_{\infty}$ output-feedback controller for a class of discrete-time T-S fuzzy systems with channel fadings, sector nonlinearities, ROIDs, and RONs. To design the controller and complete the proof of Theorem 2, *P* and $P^{-1}$ are in the form of (∗), which can be found in [7, 25]. The conditions as regards *P* in [7] are more conservative than ours because one not only needs $P>0$, but also $S>0$ and $X_{22}>0$. Therefore, Theorem 2 has less conservatism.

### Remark 6

In Theorem 2, the sufficient conditions involved in the randomly occurring nonlinearities, the probabilistic interval delays, sector nonlinearities, and channel fadings were first established for the desired fuzzy output-feedback controller. The fuzzy output-feedback controller is designed such that the discrete-time system (12) is exponentially mean-square stable and, under the zero-initial condition, the proposed $H_{\infty}$ performance index can be satisfied. Particularly, with the designed of $H_{\infty}$ fuzzy controllers, the robustness of our developed controller operation algorithms of the discrete-time fuzzy system includes the traditional controller algorithms. In other words, the traditional controller algorithms means that we have the membership function $h_{i}=1,h_{j}=0$ ($i\neq j$, $j=1,\ldots i-1,i+1,\ldots,r$) in the discrete-time fuzzy system. Obviously, the developed controller algorithms work better than the traditional algorithms in dealing with the occurrence probability of randomly occurring nonlinearities, interval delays, sector nonlinearities, and channel fadings, which appropriately avoid the deterioration of the $H_{\infty}$ performance.

## Numerical example

In this section, we present illustrative examples to show the effectiveness of the proposed controller design approach.

Consider the following discrete-time T-S fuzzy model from (10):

$$\left \{ \textstyle\begin{array}{@{}l} x(k+1)=\sum_{i=1}^{2}h_{i}\{A_{1i}x(k)+A_{2i}\sum_{m}^{2}\beta_{m}(k)x(k-\tau _{m}(k))+B_{i}u(k)\\ \hphantom{x(k+1)=}{}+D_{1i}\omega(k)+r(k)f(x(k))\},\\ z(k)=\sum_{i=1}^{2}h_{i}\{E_{i}x(k)+D_{2i}\omega(k)\}. \end{array}\displaystyle \right . $$

Consider the model parameters as follows:

$$\begin{aligned}& A_{11}= \begin{bmatrix} 0.88 & -0.55 \\ 0.31 & -0.67 \end{bmatrix} ,\qquad A_{12}= \begin{bmatrix} 0.9 & -0.65 \\ 0.312 & -0.7 \end{bmatrix} , \\& A_{21}= \begin{bmatrix} 0.103 & 0 \\ 0.2 & -0.02 \end{bmatrix} ,\qquad A_{22}= \begin{bmatrix} 0.13 & 0 \\ 0.2 & -0.02 \end{bmatrix} , \\& B_{1}=B_{2}=[ \textstyle\begin{array}{@{}c@{\quad}c@{}} 0.8 & 0.65 \end{array}\displaystyle ]^{T}, \\& D_{11}=D_{12}=[ \textstyle\begin{array}{@{}c@{\quad}c@{}} 0.22 & 0.15 \end{array}\displaystyle ]^{T}, \\& D_{21}=D_{22}=0.11, \\& E_{1}=E_{2}=[ \textstyle\begin{array}{@{}c@{\quad}c@{}} -0.96 & 0.47 \end{array}\displaystyle ], \end{aligned}$$

with the initial value $\phi(s)=[0,0]^{T} $ ($s=0, -1, -2, -3, -4, -5$). The nonlinear vector-valued function $\mathfrak{h}(k)$ is as follows:

$$\mathfrak{h}\bigl(x(k)\bigr)= \begin{bmatrix} \frac{0.06x_{1}(k)}{x_{2}^{2}(k)+1}\\ 0.015x_{2}(k)\sin(x_{1}(k)) \end{bmatrix} . $$

Besides, it can easily be noticed that Assumption 1 satisfies $\lambda =0.01$ and

$$G= \begin{bmatrix} 0.03 & 0 \\ 0 & 0.03 \end{bmatrix} , $$

the sensor nonlinearity is given as

$$g\bigl(x(k)\bigr)=\frac{M_{1}+M_{2}}{2}x(k)+\frac{M_{2}-M_{1}}{2}\sin\bigl(x(k)\bigr), $$

where

$$M_{1}= \begin{bmatrix} 0.3 & 0 \\ 0 & 0.4 \end{bmatrix} ,\qquad M_{2}= \begin{bmatrix} 0.6 & 0 \\ 0 & 0.5 \end{bmatrix} . $$

In the meantime, the output measurement is described as follows:

$$\left \{ \textstyle\begin{array}{@{}l} y(k)=g(x(k)),\\ \xi(k)=\alpha_{0}(k)y(k)+\sum_{l=1}^{2}\alpha_{l}(k)y(k-l)+\nu(k). \end{array}\displaystyle \right . $$

Here, the order of channel fading is $\ell=2$, the mathematical expectations of the channel coefficients are $\bar{\alpha}_{0}=0.9, \bar {\alpha}_{1}=0.4$ and $\bar{\alpha}_{2}= 0.2$, and the variances of the channel coefficients are $\tilde{\alpha}_{0}^{*}=0.25,\tilde{\alpha}_{1}^{*}=0.64$, and $\tilde{\alpha}_{2}^{*}=0.49$. Assume that $h=2$ for the time-varying delays, $\tau_{1}(k)$ and $\tau _{2}(k)$ are, respectively, uniformly distributed in the intervals $[2,4]$ and $[4,6]$, and the stochastic variables $\bar{\beta }_{1}=0.6,\bar{\beta}_{2}=0.3$. Other stochastic variables are $\bar {r_{1}}=0.1,\tilde{r_{1}}=0.09$.

To further illustrate the effectiveness of the designed $H_{\infty}$ fuzzy controller, the exogenous disturbance inputs $\nu (k),\omega(k)$ are assumed to be

$$\nu(k)=\frac{0.02\sin(k)}{k},\qquad\omega(k)=\frac{0.01\sin(k)}{k}. $$

**Figure 1:**
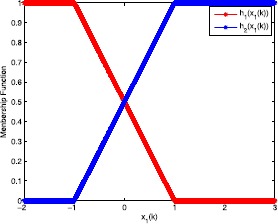
**Membership function.**

The membership functions are shown in Figure 1. The formulated T-S fuzzy model is an approximation of the original nonlinear model has been verified in [38]. In Section 2, we saw that the premise viable space can be divided into two regions from the partition method, as shown in Figure 1.

Applying Theorem 2 and the LMI toolbox, we can obtain the desired controller parameter matrices in the form of (11) such that the fuzzy system (12) is exponentially mean-square stable with the $H_{\infty}$ norm bound $\gamma=11.1$ as follows:

$$\begin{aligned}& A_{c1}= \begin{bmatrix} 0.6120 & 0.5065 \\ -0.6090 & -0.5070 \end{bmatrix} ,\qquad A_{c2}= \begin{bmatrix} 0.5531 & 0.5134 \\ -0.5082 & -0.4854 \end{bmatrix} , \\& B_{c1}= \begin{bmatrix} -2.8172&-1.5962 \\ 2.0929&3.4674 \end{bmatrix} ,\qquad B_{c2}= \begin{bmatrix} -2.7698&-1.4386 \\ 1.7566&3.2965 \end{bmatrix} , \\& C_{c1}=[ \textstyle\begin{array}{@{}c@{\quad}c@{}} 0.0034&-0.0045 \end{array}\displaystyle ],\qquad C_{c2}=[ \textstyle\begin{array}{@{}c@{\quad}c@{}} 0.0032&-0.0038 \end{array}\displaystyle ]. \end{aligned}$$

**Figure 2:**
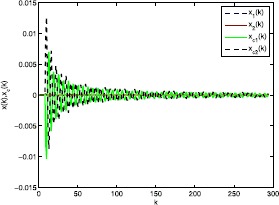
**The states of the system and the fuzzy controller.**

**Figure 3:**
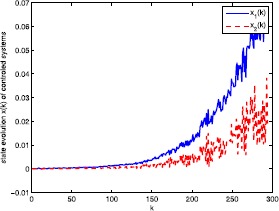
**State evolution** $\pmb{x(k)}$ **of uncontrolled fuzzy system.**

**Figure 4:**
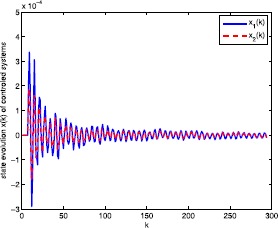
**State evolution** $\pmb{x(k)}$ **of controlled fuzzy system.**

The simulation results are shown in Figures 2-4 where the states of the system and the fuzzy controller are shown in Figure 2. We can conclude that although the discrete-time fuzzy system and the full-order output-feedback controller are subject to RONs, ROIDs, and channel fadings as well as sector nonlinearities, respectively, the fuzzy controller can estimate the state well. Moreover, we can conclude that the designed $H_{\infty}$ fuzzy filter ensures the exponentially mean-square stable of the filtering error and obtains $H_{\infty}$ disturbances rejection level *γ*. Figure 3 shows the results of the uncontrolled fuzzy system, which are clearly unstable. Figure 4 shows the consequence of the closed-loop fuzzy system, which is indeed exponentially mean-square stable. All the simulation results have confirmed that the designed $H_{\infty}$ fuzzy output-feedback control performs very well.

## Conclusions

In this paper, a fuzzy $H_{\infty}$ output-feedback controller has been designed for a class of fuzzy discrete-time systems with sector nonlinearities, channel fadings, randomly occurring interval delays as well as randomly occurring nonlinearities. A sufficient condition for the $H_{\infty}$ robust exponential stability of the fuzzy discrete-time system has been obtained by a Lyapunov stability analysis approach and stochastic analysis theory. Moreover, by using the LMI technique, a clear expression of the desired $H_{\infty}$ fuzzy output-feedback controller can be obtained and the proposed $H_{\infty}$-norm bound constraint has been guaranteed. At last, the developed fuzzy controller design approach has been checked by a numerical simulation example. Further research topics might include the development of our results to more complex and more varied cases with sector nonlinearities and channel fadings by using a stochastic analysis approach, such as multi-agent systems based on the T-S fuzzy model, descriptor systems, and affine fuzzy systems.
